# Challenges of Transitional Care for Young Allergy and Asthma Patients From Healthcare Professionals' Perspectives

**DOI:** 10.1002/clt2.70088

**Published:** 2025-08-04

**Authors:** Maria Ödling, Birgitta Lagercrantz, Susanne Lundin, Hanna Sandelowsky, Christer Janson, Inger Kull

**Affiliations:** ^1^ Department of Clinical Science and Education Södersjukhuset Karolinska Institutet Stockholm Sweden; ^2^ Department of Women's and Children's Health Karolinska Institutet Stockholm Sweden; ^3^ Sachs' Children and Youth Hospital Stockholm Sweden; ^4^ Academic Primary Healthcare Centre Stockholm Sweden; ^5^ Department of Neurobiology Care Sciences and Society Division of Family Medicine and Primary Care Karolinska Institutet Stockholm Sweden; ^6^ Department of Medical Sciences: Respiratory Allergy and Sleep Research Uppsala University Uppsala Sweden

**Keywords:** adolescent, clinical care, healthcare, transfer, transition

## Abstract

**Objective:**

Evidence‐based guidelines for transitional care of adolescents and young adults with allergy and asthma have been published, but shortcomings in the transition process have been identified. This study's aim was to gather deeper insights into healthcare professionals' experiences regarding the challenges of transitional care for adolescents and young adults with allergy and asthma.

**Methods:**

Clinically active physicians and registered nurses in Sweden were recruited through a web survey. Qualitative data were obtained through individual interviews (*n* = 18). The transcribed interview data, serving as the basis for this study, were analysed using systematic text condensation.

**Results:**

Four categories emerged during the analysis process: (1) ‘There is no clear structured approach that can be applied to everyone’, addressing variations in disease and individual patient needs. (2) ‘Relying only on referrals’, indicating that no other forms of communication exist. (3) ‘Tackling patients’ assumptions and verifying their knowledge’, referring to discrepancies between patients' expectations of healthcare professionals' knowledge of medical histories and the limited information actually available to healthcare professionals. (4) ‘Unprepared patients in a fragmented care chain’, implying a need to prepare patients for how healthcare services can differ.

**Conclusions:**

Based on healthcare professionals' experiences, transitional care for patients with allergies and asthma is a complex process marked by numerous challenges. Interventions for improvement should include increased guideline implementation at all levels of healthcare, better communication between healthcare professionals, and continuous patient education, including teaching adolescents and young adults to take responsibility for navigating the healthcare system for care of allergy and asthma.

**Abbreviations**
EAACI: European Academy of Allergy and Clinical Immunology

## 1 Introduction

In Sweden, most adolescents and young adults with allergy and asthma receive their care and treatment in primary care, whereas those with severe allergy or asthma or multiple conditions receive care in specialized paediatric outpatient clinics [1]. For those with allergy and asthma, the adolescent period means increased responsibility for self‐management (a transition), regardless of level of care. For patients in paediatric care, this period also involves a transfer to another healthcare provider around their 18th birthday [2, 3, 4]. This transitional period involves significant changes in healthcare management for young allergy and asthma patients.

Previous studies have shown that the transfer from paediatric to adult healthcare is often haphazard, for example, when the transition has not been planned appropriately [5, 6, 7, 8]. Recent publications from our group show that asthma‐related healthcare consultations were fewer than recommended in guidelines and decreased even more after the transfer to adult healthcare [9]. Also, dispensations of asthma medications decreased, even for adolescents and young adults with severe asthma. Moreover, young adults with asthma felt left out of the system and did not know where to turn in adult healthcare settings [10].

A few years ago, both the European Academy of Allergy and Clinical Immunology (EAACI) [11] and the Swedish Paediatric Society [12] published guidelines with evidence‐based recommendations for healthcare professionals to support transitional care for adolescents and young adults with allergy and asthma. However, in our web survey, deficiencies in adherence to these guidelines were identified among healthcare professionals [13]. Challenges like lack of resources, knowledge and time, cultural differences and inadequate communication between paediatric and adult healthcare settings, as well as unclear routines for management during the transition process, were mentioned. To understand the reasons behind this, the aim of this study was to conduct a qualitative investigation to gain deeper insights into healthcare professionals' experiences regarding the challenges of transitional care for adolescents and young adults with allergy and asthma.

## 2 Methods

### 2.1 Study Design and Study Population

A qualitative descriptive approach was applied to analyse the interviews, and systematic text condensation in accordance with Malterud [14] was used as a framework during this process. With the aim of including healthcare professionals who had experience of clinical work with adolescents and young adults with allergy and asthma, a purposive sampling was used [15]. Participants were recruited among 245 healthcare professionals who had previously responded to our web survey about transitional care for adolescents and young adults with allergy and asthma. All these respondents were members of the Swedish Paediatric Society for Allergy and Lung Medicine, the Swedish Primary Care Respiratory Group, the Asthma, Allergy and COPD Nursing Association, and the Swedish Association for Allergology. At the end of the web survey, they were asked whether they wanted to be part of a complementary interview study. In total, 33 individuals within paediatric care, primary care and adult allergy/asthma care answered that they wanted to be interviewed, which was found to be an appropriate sample size during planning [16]. The second author (B.L.) contacted these individuals in order to invite them to participate and book a suitable time for them to be interviewed.

### 2.2 Data Collection

Data were obtained through individual semi‐structured interviews, a method chosen to gain rich and varied data to cover the aim of the study [15, 16]. All interviews were performed during 2023 by B.L., S.L. and M.Ö., who all are registered nurses with clinical experience of working with adolescents and young adults with allergy and asthma. There was no professional relationship between the interviewers and those interviewed. The interviews were conducted in Swedish and were held in person by video link, for geographical reasons. The interviews were audio recorded and ranged in length from 25 to 45 min. To guide sample size, we used the concept ‘information power’, which indicates that the more information a sample holds, the fewer participants are needed [16]. It is suggested that what is sufficient information power depends on the aim of the study, the sample specificity, the use of established theory, the quality of dialogue and the analysis strategy. In the present study, factors that had an impact on information power were the narrow aim, the relevant and purposive sample of participants, the focused quality of the dialogue, and the chosen analysis strategy.

The interview guide was developed based on joint discussions between the authors about factors of importance in the interviews, using previous knowledge as professionals with clinical experience of allergy and asthma management [17]. A preliminary interview guide was draughted and pilot tested. The final, slightly revised, interview guide is presented in the Supporting Information S1. Supplementary follow‐up questions were asked for clarification purposes if necessary.

The researchers received information from the Swedish Ethical Review Authority that ethical approval was not needed for this study. At the beginning of the interviews, the respondents got verbal and written information on how the material would be treated, the aim of the study and that their participation was voluntary, and they gave written informed consent.

### 2.3 Data Analysis

**FIGURE 1:**
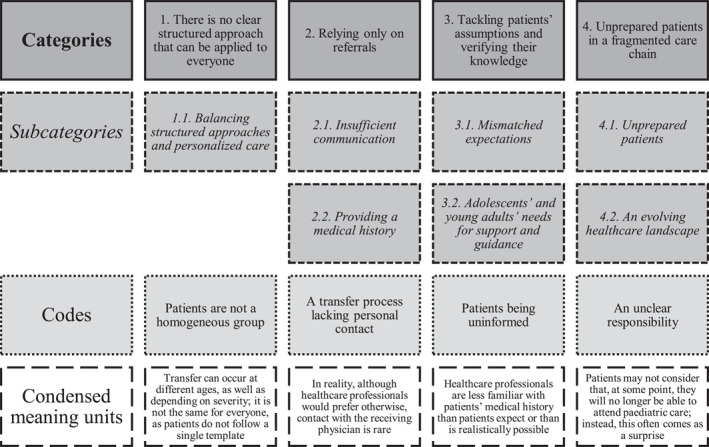
Categories, subcategories, codes and condensed meaning units.

In accordance with the concept ‘information power’, the sufficiency of the final sample size was evaluated continuously during the research process, resulting in a total of 18 healthcare professionals being included in the final analyses. Recordings were transcribed verbatim, and the transcribed data were analysed using systematic text condensation. Systematic text condensation is a descriptive approach, presenting the experiences of the respondents, as expressed by themselves. Systematic text condensation entails intersubjectivity, reflexivity, feasibility, and a responsible level of methodological rigour [10, 14]. We visualized the categories, subcategories, codes and condensed meaning units in the analyses in Figure 1.

To achieve credibility in the analytical process, all steps were first conducted by M.Ö. and I.K. separately. The two authors discussed their results jointly until consensus was achieved [18], with all authors engaging in discussions about the emerging findings until agreement was reached. Relevant quotations from the interviews were chosen to highlight the findings and professionally translated into the English language.

## 3 Results

The study population consisted of 10 nurses and eight physicians (Table 1). The great majority were experienced in their profession: all but one (94%) had worked for more than 11 years. In total, five (28%) worked in paediatric care, six (33%) in primary care and seven (39%) in adult allergy/asthma care.

**TABLE 1:** Characteristics of interviewed respondents (*n* = 18).

Respondent #	Sex	Workplace	Profession	Years in profession
1	Female	Paediatric care	Nurse	> 11
2	Female	Adult allergy/asthma care	Nurse	> 11
3	Male	Primary care	Physician	> 11
4	Female	Primary care	Nurse	> 11
5	Male	Adult allergy/asthma care	Physician	> 11
6	Female	Paediatric care	Nurse	> 11
7	Female	Paediatric care	Nurse	> 11
8	Female	Adult allergy/asthma care	Nurse	> 11
9	Female	Primary care	Physician	> 11
10	Female	Adult allergy/asthma care	Physician	> 11
11	Female	Adult allergy/asthma care	Nurse	> 11
12	Female	Primary care	Nurse	> 11
13	Male	Adult allergy/asthma care	Physician	> 11
14	Male	Primary care	Physician	6–10
15	Female	Paediatric care	Physician	> 11
16	Female	Adult allergy/asthma care	Nurse	> 11
17	Female	Paediatric care	Nurse	> 11
18	Male	Primary care	Physician	> 11

With regard to healthcare professionals' views on experiences regarding the challenges of transitional care, four categories and seven subcategories emerged during the analysis process. The categories were: (1) ‘There is no clear structured approach that can be applied to everyone’, (2) ‘Relying only on referrals’, (3) ‘Tackling patients’ assumptions and verifying their knowledge’, and (4) ‘Unprepared patients in a fragmented care chain’ (Figure 1).

### 3.1 There Is No Clear Structured Approach That Can be Applied to Everyone

The healthcare professionals acknowledged the value of structured approaches. However, they highlighted that patients do not follow a uniform template and there was no one‐size‐fits‐all solution due to variations in disease and individual patient needs.

#### 3.1.1 Balancing Structured Approaches and Personalized Care

The healthcare professionals stated that they recognized the importance of structured approaches to adolescents' and young adults' transitions and transfers. However, they also said that it was common to treat all patients in the same manner, even though the disease can vary as regards both severity and individual patient needs.

Be a bit clearer about what the kids need to learn and when they might be ready for it, because I don’t feel like we have a good work method, but that’s because it’s always different, and of course it has to be different for them.

(no 7)

### 3.2 Relying Only on Referrals

It was described that beyond referrals, which did not always include a complete medical history, there was little other communication between centres. This limited interaction was further complicated by the fact that centres sometimes used different electronic health record systems, which meant they could not access each other's clinical notes.

#### 3.2.1 Insufficient Communication

The healthcare professionals stated that the primary mode of communication with primary care was through referrals, resulting in limited access to clinical notes. This was further complicated by the use of different electronic medical record systems.

Aside from referrals, we don’t really have any other communication, it’s really just communication through referrals. We have different medical record systems, so it’s not even a given that we can access their notes and they can basically never access our notes, so what we found out about each other’s patients is really just through referrals.

(no 14)

Moreover, a reliance on referral information for patient follow‐up was described. The healthcare professionals typically sent a referral to the primary care centre where an adolescent or young adult was registered for healthcare, and their involvement essentially ended there. The referring healthcare professionals could only hope that they would take care of the patient thereafter.

We really just send a referral to the primary care centre where the kid has its healthcare contact and then we’re not involved in that anymore. It’s just … send the referral and hope that they take care of them.

(no 7)

#### 3.2.2 Providing a Medical History

The receiving healthcare professionals stated that there were clear deficiencies in many referrals. They wanted to get a summary and information about what had been planned by the referring provider going forward or what had been discussed with the patient.

There’s just a referral with two or three lines and a bunch of medical record notes. That should be improved. What you want is a summary and what they have imagined going forward from paediatric care and what has been discussed with the child or young adult.

(no 9)

### 3.3 Tackling Patients' Assumptions and Verifying Their Knowledge

The healthcare professionals recognized a discrepancy between patients' expectations of their knowledge of the patients' medical history and the limited information actually available to them. Moreover, the healthcare professionals emphasized the need to verify each adolescent's or young adult's capabilities and understanding, rather than assuming the patient would take full responsibility or would have full knowledge.

#### 3.3.1 Mismatched Expectations

The receiving healthcare professionals believed that patients assumed they were much more familiar with each patient's entire medical history than they were. Further, the receiving healthcare professionals stated that they normally did not have a sufficient understanding of the background of a new patient. Consequently, the care delivered by the new healthcare professionals do not always meet the patients' expectations.

I think they think we know much more about their background, like that they can mentioned anything and they think we’ll have full insight into everything in their history, which we don’t in this way. So, I think that maybe they expect much more than we have the ability to provide.

(no 5)

#### 3.3.2 Adolescents' and Young Adults' Needs for Support and Guidance

The healthcare professionals stated that they might need to be more attentive to and understanding of the fact that adolescents and young adults might not yet be fully capable of self‐management, or at the very least, might need to verify with a young patient before expecting them to take full responsibility for themselves. The healthcare professionals needed to ensure that adolescents and young adults were knowledgeable and capable, rather than simply assuming they knew precisely how to act in their contacts with the healthcare system.

That we in adult healthcare maybe need to be more perceptive and understand that eighteen‐ or nineteen‐year‐olds can’t, or that at least we need to check, before we expect them to take full responsibility on their own, we need to check that they know, that they are able. We can’t just assume that they know precisely how to act in the contacts with healthcare.

(no 10)

### 3.4 Unprepared Patients in a Fragmented Care Chain

The healthcare professionals stressed the need to prepare patients for the transfer to adult healthcare, suggesting more proactive approaches. Moreover, they stated that the deficiencies in the care chain impeded effective collaboration among various healthcare providers.

#### 3.4.1 Unprepared Patients

The respondents stated that the referring healthcare professionals could involve and prepare patients by asking which primary care centre they were registered at and explain the process of care there. In particular, it was pointed out that the referring healthcare professionals needed to teach the adolescents and young adults to be more active and take more responsibility for their care navigation than what they have been used to in paediatric healthcare. This included explaining that no one would ‘chase’ an adult patient if they missed an appointment.

That we have, like prepared, if we have asked what primary care centre they are registered at and have told them how it will work and I’ve probably told them that they need to be maybe a bit more active, like that no one will chase them if they don’t come to their appointments. No, so I think we’ve made some good progress there.

(no 1)

#### 3.4.2 An Evolving Healthcare Landscape

The healthcare professionals described challenges in determining post‐transfer patient care responsibility, citing the complex network, and the continuous emergence of new healthcare centres as well as frequent staff turnover. The scarcity of specialized clinics accommodating adolescents and young adults compounded this issue, resulting in limited care options and hindering effective collaboration.

The problem with primary care is that you often don’t know who will be taking over, as there are so many units and different staff and so on.

(no 7)

## 4 Discussion

This qualitative study aimed to explore the underlying reasons for deficiencies in the management in transitional care for adolescents and young adults with allergy and asthma in Sweden. The four categories—(1) ‘There is no clear structured approach that can be applied to everyone’, (2) ‘Relying only on referrals’, (3) ‘Tackling patients’ assumptions and verifying their knowledge’ and (4) ‘Unprepared patients in a fragmented care chain’—provided important knowledge of healthcare professionals' perspectives regarding the challenges of transitional care including the unclear routines, lack of resources, inadequate communication, gaps in healthcare professionals' knowledge, and cultural differences between paediatric and adult healthcare settings.

In the present study, the healthcare professionals underlined the importance of structured approaches for adolescents' and young adults' transitional care, but said that, in practice, using a person‐centred methodology was challenging due to the variability in disease severity and individual patient needs. This discrepancy between recognized best practices and actual work may explain the unclear routines observed in the management of the transition process. A recent review [19] argues that current guidelines and recommendations, including the Global Initiative for Asthma annual report [20] and the EAACI guideline on effective transition [11], emphasize the need to establish and implement transition programmes for adolescents and young adults with allergy and asthma. However, these recommendations lack specific details on the structural components, contents and key elements of such transition programmes. The transitional care requirements for adolescents and young adults with allergy and asthma may vary significantly, highlighting the need for personalized approaches in transition programme development. This was also evident in our study. Following on the publication of the EAACI guideline [11], a ‘toolbox’ paper has been published [2], including a selection of practical resources to aid implementation of the guideline and assist the multiple centres and healthcare professionals involved in transition.

It is well‐known that a well‐designed transition service necessitates collaboration between paediatric and adult allergy and asthma teams through regular joint meetings, shared protocols, and effective communication [21]. However, in our study, the healthcare professionals described challenges in communication and patient transfer between centres due to the fragmented healthcare structure, personnel turnover and limited specialized clinics for adolescents and young adults. Communication was largely limited to referrals, with minimal additional interaction, and sometimes hindered by the use of different medical record systems. Although Swedish healthcare may differ from that in other countries, a recent European survey revealed similar challenges reported by healthcare professionals, with nearly half indicating a lack of an established feedback system between paediatric and adult medical services following the transfer of care for adolescents and young adults [22]. Additionally, only about 10% reported that a medical report was sent from the adult clinic to the referring paediatrician, and 9% reported discussing patients at regular meetings between services.

In our study, the healthcare professionals acknowledged that adolescents and young adults need to be prepared for how healthcare services differ between paediatric and adult settings, including the increased emphasis on personal responsibility in adult healthcare, highlighting the pressing need to prepare adolescents and young adults for self‐management [11]. For example, in Sweden, adult patients are generally not automatically called for follow‐up appointments. Instead, they are expected to take responsibility for booking their own check‐ups, a task they must manage independently as young adults, unlike when they were children. A previous systematic review of qualitative and quantitative studies investigating barriers to and facilitators of asthma self‐management concluded that current clinical practice should focus on ensuring that adolescents have the requisite knowledge and skills to self‐manage their disease [23]. This was also found to be important for adolescents and young adults and their parents when the EAACI guidelines were evaluated [24]. The most supported recommendation was checking that the adolescents and young adults had knowledge of and were adherent to their prescribed medication. In addition, care should be provided in a supportive environment that facilitates two‐way communication and fosters an adolescent's or young adult's self‐efficacy in managing their disease. This can be essential for their being able to manage treatment plans and improve their health outcomes [25].

In the present study, healthcare professionals in adult healthcare settings reported a discrepancy between patients' expectations of healthcare professionals' knowledge of their medical history and the actual information available, emphasizing the need for both referring and receiving healthcare professionals to verify a patient's understanding and capabilities during the transition process, rather than assuming full patient autonomy. This discrepancy may be explained by the cultural differences between paediatric and adult healthcare settings. It may also explain the high demands on healthcare professionals' knowledge of managing adolescents and young adults with allergy and asthma during transitional care. A recent EAACI task force report on the global assessment of the knowledge and confidence in managing allergic disorders among primary care paediatricians across Europe highlighted the importance of allergy training, possibly starting as early as undergraduate studies, to reach all future primary care professionals [26]. It also supported the importance of harmonizing allergy care and promoting a multidisciplinary approach through a network of specialists and primary care professionals.

## 5 Strengths and Limitations

Several strengths and limitations in our study should be acknowledged. Through the purposive sampling, credibility was promoted, as the respondents provided sufficient variety to highlight the purpose, with aspects such as level of care and profession considered during recruitment to ensure a full range of perspectives of transitional care management [15]. We acknowledge that the small sample may have constrained our ability to detect profession‐specific patterns within the data. However, the aim of our study was to gain in‐depth insights into healthcare professionals' experiences, which is well supported by a qualitative approach. If the research objective were instead to examine statistically significant differences between professional groups, the use of quantitative methods would be more appropriate, as these allow for statistical comparisons and more generalizable conclusions across larger samples.

The sample size was based on ‘information power’ [16], and overall, we believe that our study achieved sufficient information power.

In the present study, all authors were involved in the care of adolescents and young adults with allergy and asthma and therefore needed to be aware of their pre‐understanding and bracket this in as far as possible. However, given that the authors had different professional and clinical experiences of involvement, the pre‐understandings could be bracketed both in the interview situation and in the analysis process. Although qualitative research is not generalizable, it provides rich insights and a comprehensive understanding of complex phenomena that can contribute to clinical practice. However, despite current guidelines and recommendations regarding the transition process, the absence of a structured, approach across settings, identified as a category in our results, also limits the broader applicability and comparability of our study. In addition, the findings of this study may not be transferable beyond the Swedish healthcare context, and the fragmented nature of the sample further limits both transferability and analytical depth.

## 6 Conclusions

Based on the healthcare professionals' experiences, transitional care for patients with allergies and asthma in the Swedish healthcare system appears to be a complex process marked by numerous challenges. Interventions for improvement should include increased guideline implementation at all levels of healthcare, better communication between healthcare professionals, and continuous patient education, including teaching adolescents and young adults to take responsibility for navigating their care of allergy and asthma in the healthcare system.

## Author Contributions

M.Ö., B.L. and I.K. made substantial contributions to the design of the work. M.Ö. and I.K. analysed and interpreted the data. M.Ö. was a major contributor in writing the manuscript. I.K. substantively revised the work. All authors read and approved the final manuscript.

## Conflicts of Interest

The authors declare no conflicts of interest.

## Supporting information

Supporting Information S1

## Data Availability

The datasets generated during the present study are available from the corresponding author on reasonable request.
