# Supplementary material for: Challenges of Transitional Care for Young Allergy and Asthma Patients From Healthcare Professionals' Perspectives
Source: Clin Transl Allergy. 2025 Aug 4;15(8):e70088. doi: 10.1002/clt2.70088 (PMC12321966; doi:10.1002/clt2.70088)
Supplement: Supplementary file 1 — Supporting Information S1 [file CLT2-15-e70088-s001.docx]

**Supporting Information**

**Challenges of transitional care for young allergy and asthma patients from healthcare professionals’ perspectives**

Maria Ödling, Ph.D., Birgitta Lagercrantz, M.Sc., Susanne Lundin, Ph.D., Hanna Sandelowsky, Ph.D., Christer Janson, Ph.D., & Inger Kull, Ph.D.

**Interview guide**

**Main questions**

1. With a focus on transition and transfer of patients with asthma and/or allergy, can you tell me in general which of these patients you have at your workplace?
2. Do you have any examples of when you have discussed the transition from child to adult, i.e., the development-related transition in age/maturity, at your workplace?
3. Tell me what transfer, i.e., the actual event in the form of switching from a care provider in paediatric healthcare to adult healthcare/before and after the age of 18 years, means to you?
4. Can you describe your thoughts on the transfer from paediatric to adult healthcare?
5. Can you give examples of how you/your workplace prepares patients for a transfer?
6. Can you tell me more about how you perform transfers at your workplace?
7. Can you tell me about the distribution of responsibilities in transfer from your workplace to another care provider?
8. Can you tell me some more about how you perceive the expectations of the adolescents/young adults?
9. Is there anything else you want to mention regarding the transition or transfer, that we haven’t discussed already?
